# Sleep duration is associated with vitamin D deficiency in older women living in Macao, China: A pilot cross-sectional study

**DOI:** 10.1371/journal.pone.0229642

**Published:** 2020-03-04

**Authors:** Xiaoying Liu, Liang Ke, Jacky Ho, Myriam Abboud, Elias Mpofu, Tara C. Brennan-Speranza, Rebecca S. Mason, Kaye E. Brock

**Affiliations:** 1 Department of Physiology, School of Medical Sciences, Bosch Institute, The University of Sydney, Sydney, Australia; 2 Macao Hypertension League, Macao, China; 3 City University of Macao, Macao SAR, China; 4 Zayed University, Dubai, UAE; 5 Clinical and Rehabilitation Sciences Research Group, Faculty of Health Science, The University of Sydney, Sydney, Australia; 6 Rehabilitation and Health Services, University of North Texas, Denton, TX, United States of America; 7 Educational Psychology, University of Johannesburg, Johannesburg, South Africa; 8 School of Public Health, Faculty of Medicine and Health, University of Sydney, Sydney, Australia; Arizona State University, UNITED STATES

## Abstract

Chinese women are known to have both a high prevalence of metabolic syndrome (MetS) and vitamin D deficiency (serum 25-hydroxyvitamin D (25OHD) <50 nmol/l). Associations between sleep duration and circulating 25OHD have recently been reported but, to our knowledge, these associations have not been studied in older Chinese populations. We thus investigated whether sleep duration was associated with vitamin D status in a population from Macao, China, and whether sleep duration modified the association between MetS and vitamin D deficiency. In 207 older (>55 years) Macanese, anthropometry, blood samples and validated questionnaires, including sleep duration and cardiovascular risk factors, were simultaneously collected. On multivariable categorical analyses, those women, not men, who had short sleep duration (≤6 hours (h)) were at a 2-fold risk for vitamin D deficiency (both <50 nmol/L and <37 nmol/L; OR = 1.94, 95%CI 1.29–2.92; OR = 2.05, 95%CI 1.06–3.98, respectively) and those who had longer sleep duration (>8 h) were 3-fold more likely to have vitamin D deficiency (OR = 3.07, 95%CI 1.47–6.39; OR = 2.75, 95%CI 1.08–7.00, respectively) compared to those with normal sleep duration (6–8 h). Both women and men with MetS were 2-fold more likely to have vitamin D deficiency (women: OR = 2.04, 95%CI 1.31–3.17; OR = 2.15, 95%CI 1.11–4.17, respectively; men: OR = 2.01, 95%CI 1.23–3.28; OR = 2.04, 95%CI 1.00–4.29, respectively). Moreover, women with both short sleep duration and MetS had an increased risk of vitamin D deficiency (OR = 3.26, 95%CI 1.10–9.64). These associations were not found in those with longer sleep. Men with longer sleep and MetS had a 5-fold risk of vitamin D deficiency (OR = 5.22; 95%CI 2.70–10.12). This association was non-significant for men with shorter sleep. We conclude that both short and long sleep duration were associated with vitamin D deficiency in older Chinese women. Further research is needed in larger cohorts or with intervention studies to further examine the associations between reduced sleep, metabolic syndrome and vitamin D deficiency.

## Introduction

Vitamin D is a hormone precursor, necessary for facilitating the absorption of calcium and phosphate ions from the gut [1]. Vitamin D deficiencies lead to osteomalacia, or rickets in children, both of which are problems of impaired bone mineralization. More recent studies have also indicated that vitamin D is beneficial for the prevention of skin cancer [2–4], and for improving muscle function and other physiological and pathophysiological processes [5–8]. In humans, following exposure to ultraviolet B radiation from the sun, 7-dehydrocholesterol is converted to Vitamin D. Vitamin D can also be obtained from the diet, however, skin production is more important in humans. Vitamin D status is defined by the amount of circulating 25-hydroxyvitamin D (25OHD). Vitamin D insufficiency (25OHD<50 nmol/L) varies across populations worldwide [9–11] and is consistently reported to be common in Chinese populations [12–26], especially in women [12, 15, 21, 22, 24, 25, 27, 28]. Investigations into the factors associated with vitamin D deficiency in Chinese women have indicated that the traditional culture of using umbrellas and/or sunscreen to avoid tanning results in a lack of sunlight exposure leading to reduced vitamin D levels [12, 27].

In addition, a recent meta-analysis of epidemiological observational (n = 849,412) and randomized intervention studies (n = 30,716) has reported an inverse association of circulating 25OHD with risk of cardiovascular (CVD) death (Odds Ratio [OR] = 1.35, 95%CI 1.13–1.61) [29] and Chinese populations are reported to have an increasingly high prevalence of CVD [30]. Other data from this same study from Macao on associations between CVD risk and vitamin D deficiency has been previously published [31]. Cardiovascular mortality is also reported to be associated with both short and long sleep duration [32]. Three recent meta-analyses reported associations between CVD mortality and sleep duration. Both short sleep duration (Relative Risk [RR] = 1.18, 0.76–1.84) and long sleep duration (RR = 1.43, 1.15–1.78) were associated with CVD mortality [33–35]. In addition, metabolic syndrome, a cluster of risk factors for CVD (central obesity, reduced high-density lipoprotein cholesterols [HDL], increased blood pressure, blood glucose and blood triglycerides [TG]), were also reported to be associated with both short and long sleep duration [36, 37].

Vitamin D deficiency is associated with sleep apnoea [38] and sleep duration, however, there is conflicting data from intervention trials on the effect of vitamin D supplementation on sleep quality in men and women [39]. To our knowledge, no study has investigated the association between sleep duration and vitamin D status in adult Chinese populations. We have previously reported associations between vitamin D and CVD risk factors in a representative population from Macao, China (n = 566) [28]. As previously noted, metabolic syndrome is a risk factor for vitamin D deficiency [36, 37]. Very few studies have examined the interaction effect of short or long sleep duration and CVD risk factor on vitamin D deficiency. We thus took the opportunity to investigate sleep duration and vitamin D deficiency and CVD risk in those age >55 years from this population (n = 207) taking into account their metabolic syndrome status. We also examined whether sleep duration modified the association between metabolic syndrome and vitamin D deficiency. As older females had a much higher rate of vitamin D deficiency compared to men (47% in female vs. 26% in male) [12], the specific aims of this pilot were to investigate the relationship between vitamin D status and short (≤6 h) or long (>8 h) sleep duration compared to normal sleep duration (6 to 8 h) [40]) in both men and women.

## Materials and methods

### Study sample and data collection

**Fig 1:**
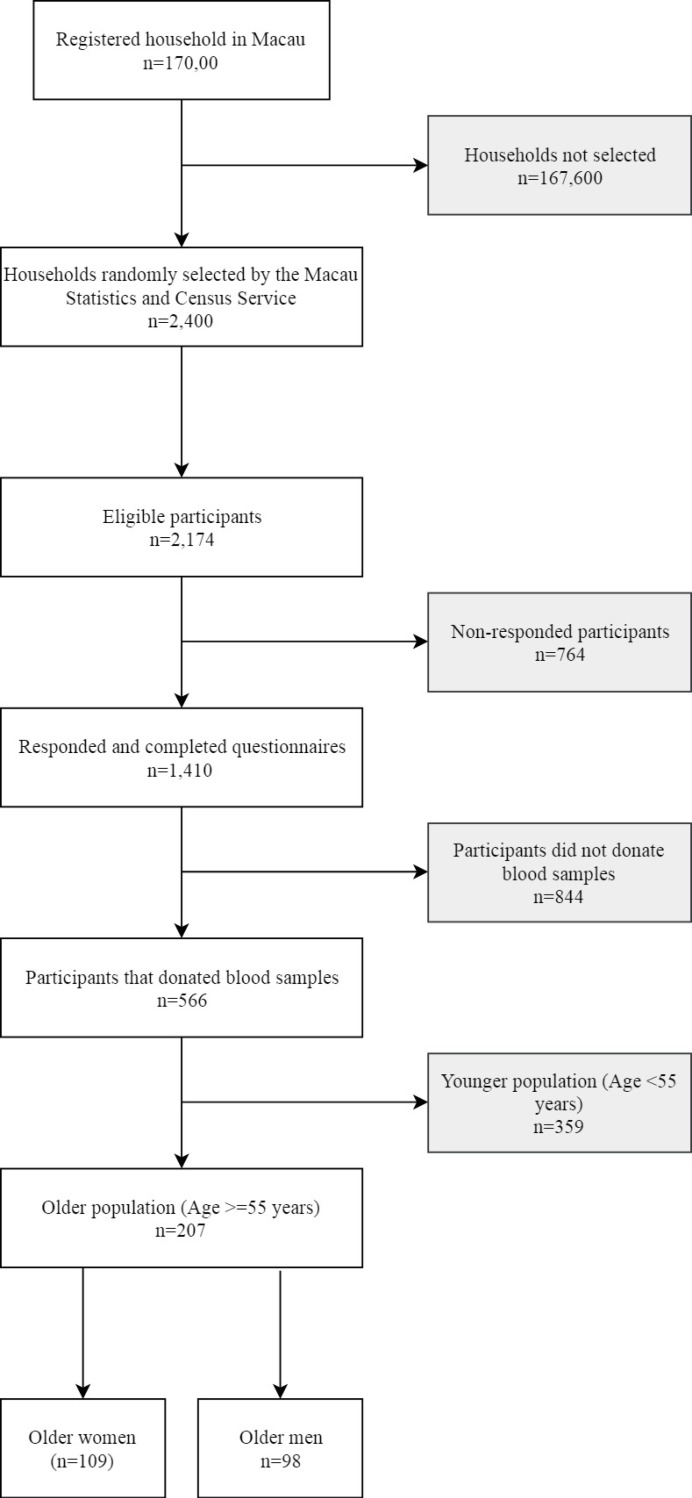
Flow diagram of study participants.

A representative population sample from Macao was investigated in 2014, detailed methods of sampling and data collection by individual measurement and validated questionnaires have previously been documented [12, 28, 41]. In brief, based on an estimated hypertension prevalence of 28% in 2012, a sample size of 1,488 was predicted; with an estimated 50% response rate, 2,400 households (approximately 2.5 members per household) were randomly selected from a register of 170,000 households. A total of 1,410 study participants from 2,174 eligible participants were recruited (response rate 68%) and had completed measurement and survey. Of these 1,410 participants, 566 participants had donated blood samples for analyzing serum 25OHD and metabolic parameters. We had reported that the prevalence of vitamin D insufficiency was 55% in the total population, and it was estimated that the prevalence of vitamin D <50 nmol/L was 45% in China [42]. The post-hoc power was 99%. In these 566 participants, thirty-seven percent were older population (n = 207; age >55 years) (Fig 1). Furthermore, two older females and one male had no data on sleep duration, therefore, only 204 participants were included in the current analysis.

Sleep duration was documented as number of hours (h) of sleep per day on weekdays and weekends. We scaled the question responses retaining the intent of the Pittsburgh Sleep Questionnaire Index item [43] making for greater precision to guide our analysis. Daily average sleep duration was then calculated = [(sleep duration in weekday*5 + sleep duration in weekend*2) / 7]. A comparison of short sleep (≤ 6h) and long sleep duration (>8 h) were compared to normal sleep duration (6–8 h). Although this normal duration differs slightly from the recommendations of the US National Sleep Foundation and American Academy of Sleep Medicine [44], the proposed 6–8 h “normal” sleep duration for this population comes from recent studies of the association between sleep duration and CVD events in Chinese population [45, 46].

Concurrently (in summer) fasting blood samples were collected and serum 25OHD was determined using an electrochemiluminescence immunoassay (Roche Diagnostics) with an intra-assay coefficient of variation of 1.93%. As opinion regarding optimal levels of vitamin D is divided [11, 47]. Chinese populations are known to have quite low mean values of vitamin D. Thus, vitamin D insufficiency was defined as serum 25OHD <50 nmol/L and vitamin D deficiency was defined as serum 25OHD <37 nmol/L in the current investigation.

Serum high-density lipoprotein (HDL) levels were determined using homogeneous enzymatic colorimetric assays and serum triglycerides (TG) were determined using an enzymatic colorimetric assay. Metabolic syndrome (MetS) was defined as the presence of any three of the following: overweight (BMI ≥24 kg/m^2^), hypertension (measured blood pressure >140/90 mmHg and/or self-reported diagnosis and/or treatment of hypertension in the last two weeks), low serum HDL (serum HLD <1.03 mmol/L) or high TG (serum TG ≥1.7 mmol/L) (National Cholesterol Education Program Adult Treatment Panel III criteria) [48].

Socioeconomic status (SES), physical activity (PA), and fish consumption data were collected from individually administrated questionnaires [12, 28, 41].

Socioeconomic status was adopted from area coding according to the income level of resident area [49].

Physical activity was measured by the International Physical Activity Questionnaire Short Version [50]. The walking, moderate and vigorous metabolic equivalent task (MET) minutes per week and the total MET minutes per week were calculated according to the IPAQ scoring protocol.

Information regarding oily fish consumption was collected by asking participants “Do you usually (≥4 times/week) eat fatty fish?”

Individual questionnaire survey and blood sample collection were conducted at the one visit. Ethics approval was obtained from both the Human Research Ethics Committee of the University of Sydney, Australia and Sun Yet-Sen University China. Informed consent was obtained in written form.

### Statistical analysis

The present sample was selected using a complex sample design where the study subjects were sampled with differential selection probabilities. Non-response and post-stratification adjustments were conducted to obtain the sample weights (the sample weights are the number of observations in the population that can be represented by the sampled observations) [51]. Survey data was weighted, and age and sex standardization was performed based on the population distribution from the Macao 2011 census, and thus, the selected sample was representative of the Macao population [41].

Data were presented as mean and standard deviation (SD) for continuous variables; and as proportions for categorical variables. Differences of population characteristics between older and younger population was calculated by chi-square test. Levels of serum 25OHD and sleep duration in those with short (≤6h) or normal (>6h and ≤8h) or long (>8h) sleep duration, and MetS were calculated using student *t*-tests. Vitamin D risk factors included SES, PA, and fish consumption [12, 28, 31] and MetS. In our conditional logistic regression model investigating vitamin D deficiency in this population, each risk factor variable (SES, PA and fish consumption) was added individually and if still significant, left in the model as a confounder.

The association between sleep duration and serum 25OHD levels was firstly assessed by quadratic regression stratified by sex. Quadratic term of sleep duration was added to the linear regression to test the quadratic association between sleep duration and serum 25OHD levels.

As an inverted ‘u-shape’ (or biphasic) relationship was observed between sleep duration and serum 25OHD levels, the associations between sleep and vitamin D deficiency were then assessed by univariable and multivariable logistic regression analyses. In detail, associations with vitamin D deficiency (as defined as both less than 37 nmol/L or less than 50 nmol/L) were then assessed by multivariable conditional logistic regression analyses, adjusting for SES, PA, and fish consumption and presented as odds ratios (OR) with 95% confidence intervals (CI). In this population from Macao, there were marked differences for predictors of vitamin D deficiency between the sexes (we previously reported significantly statistical interaction [12]); thus these data were stratified by sex.

In this current population, we also tested for multiplicative statistical interaction between sleep duration and MetS with vitamin D status. This was assessed by the likelihood ratio test in a logistic model [52] for vitamin D status, which included main effects for sleep duration (≤6h) and MetS and the interaction term between sleep duration and MetS (Yes/No). As this was highly significant (p<0.001), subsequent models were presented stratified by short (≤6h) or normal-long (>6h) sleep duration. According to our criteria, none of the older male participants with <6 h sleep had vitamin D levels <37 nmol/L fullfiled the criteria of MetS. Thus, analysis of this group was not possible, unless we classified one of the participants that was borderline for MetS as having MetS. As such, these results are based on the latter assumption.

For all analysis, statistical significance was defined as p<0.05. All data analyses were performed with SPSS 21 statistical package (IBM, Armonk, NY)

## Results

We have previously reported associations between vitamin D status and CVD risk factors in a representative population from Macao, China (n = 566) [28]. Table 1 shows the participant characteristic by age groups. Although the older population had lower prevalence of vitamin D insufficiency and severe deficiency compared to the younger population (36% vs. 63% and 10% vs. 28%, respectively), CVD risk factors, including overweight (48% vs. 34%), TG (30% vs. 20%), hypertension (61% vs. 20%) and MetS (21% vs. 9%) were higher in the older population. In addition, the rate of short sleep duration (<6 h/day) was significantly higher in the elderly than the younger (32% vs. 19%).

**Table 1:** Characteristics of Macao population (n = 566) stratified by age.

	Older (n = 207)	Younger (n = 359)
25OHD <50 nmol/L	36	63*
25OHD <37 nmol/L	10	28*
Sleep <6h /day	32	19*
BMI ≥24 kg/m^2^	48	34*
Decreased HDL	20	17
Increased TG	30	20*
Hypertension	61	20*
MetS	21	9*
Mod-high PA	79	72*
Oily fish <4 time/wk	32	28*
Low SES	35	44*
Female	53	64*

Data were presented as %

*p<0.05 compared proportion between older and younger Macanese populations

We thus investigated sleep duration and vitamin D status and CVD risk the older population from this population (n = 207). There were significant differences in vitamin D deficiency rates (serum 25OHD <50 nmol/L and <37 nmol/L) in women versus men in this population. The prevalence of serum 25OHD <50nmol/L was 47% in women vs. 26% in men (p<0.001). The prevalence of serum 25OHD <37 nmol/L was 12% in women vs. 8% in men (p<0.001). The mean serum 25OHD levels were 62.76 nmol/L for men and 54.46 nmol/L for women (p<0.001).

**Fig 2:**
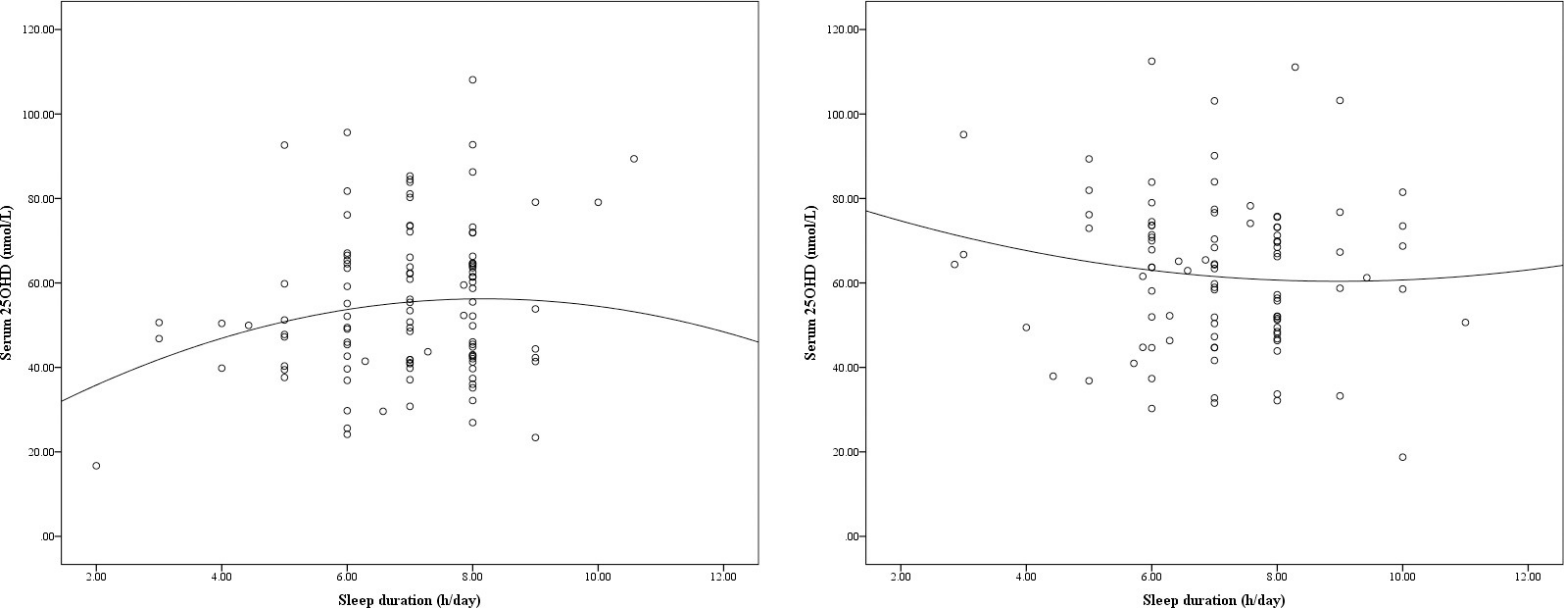
**A.** Sleep duration and serum 25OHD in older women living in Macao, China (n = 108). **B.** Sleep duration and serum 25OHD in older men living in Macao, China (n = 96).

In older Macanese females, when the association between sleep duration and serum 25OHD levels was assessed by the quadratic regression, both sleep duration and the quadratic term were significantly associated with serum 25OHD levels (y = 20.46+8.75x-0.54x^2^, p<0.05 for both coefficients; R^2^ = 0.03). As shown in Fig 2A, both long sleep duration and short sleep duration were associated with decreased serum 25OHD levels in these older Macanese women. In contrast, both sleep duration and the quadratic in older men were not associated with serum 25OHD levels (y = 84.09–5.28x+0.29x^2^; p>0.05 for both coefficients; R^2^ = 0.015; Fig 2B).

In women, on conditional regression analysis, those with short and long sleep duration were at a 2- to 3-fold increased risk of vitamin D deficiency (either <50 nmol/L or <37 nmol/L) compared to those with normal sleep (short sleep duration OR = 1.94, 95% CI 1.29–2.92 and OR = 2.05, 95% CI 1.06–3.98 respectively, and longer sleep duration OR = 3.07, 95% CI 1.47–6.39 and OR = 2.75, 95%CI 1.08–7.00 respectively) (Table 2).

**Table 2:** Sleep duration (h) and risk of serum 25OHD <50 nmol/L or 25OHD <37 nmol/L in older Macao residents (n = 204).

	%	Mean sleep duration (h)	Mean 25OHD (nmol/L)	25OHD<50 nmol/L		25OHD <37 nmol/L	
				Crude OR (95%CI)	Adjusted OR^a^ (95%CI)	Crude OR (95%CI)	Adjusted OR^b^ (95%CI)
**Women (n = 108)**							
**Sleep duration (h)**							
Sleep >6 to ≤8 (reference)	61	7.5 ± 0.5	56 ± 17	1.0	1.0	1.0	1.0
Sleep≤6	30	5.3 ± 1.0*	52 ± 17*	1.55 (1.05–2.30)*	1.94 (1.29–2.92)*	1.47 (0.80–2.70)	2.05 (1.06–3.98)*
Sleep>8	8	9.2 ± 0.5*	50 ± 23	2.25 (1.12–4.54)*	3.07 (1.47–6.39)*	2.89 (1.24–6.74)*	2.75 (1.08–7.00)*
**MetS**							
No (reference)	75	6.9 ± 1.3	56 ± 18	1.0	1.0	1.0	1.0
Yes	25	7.2 ± 1.3	50 ± 16*	1.95 (1.28–2.95)*	2.04 (1.31–3.17)*	1.55 (0.85–2.82)	2.15 (1.11–4.17)*
**Men (n = 96)**							
**Sleep duration (h)**							
Sleep >6 to ≤8 (reference)	55	7.4 ± 0.6	60 ± 15	1.0	1.0	1.0	1.0
Sleep≤6	31	5.4 ± 1.0*	65 ± 19	0.89 (0.57–1.39)	0.75 (0.48–1.20)	0.98 (0.46–2.10)	0.85 (0.39–1.84)
Sleep>8	14	9.5 ± 0.7*	65 ± 23	0.45 (0.21–1.00)	0.56 (0.26–1.21)	2.28 (0.98–5.31)	3.01 (1.22–7.42)*
**MetS**							
No (reference)	80	7.2 ± 1.5	65 ± 18	1.0	1.0	1.0	1.0
Yes	20	6.5 ± 1.4*	56 ± 15*	2.30 (1.45–3.65)*	2.01 (1.23–3.28)*	1.99 (0.99–3.99)	2.04 (1.00–4.29)*

*p<0.05 for independent t-test and logistic regression compared to the reference group

^a^ Mutually adjusted for sleep duration, MetS, SES, PA, fish consumption

^b^ Mutually adjusted for sleep duration, MetS, SES and PA; Data presented as mean +SD where appropriate

In men, shorter sleep (≤6 h) was not significantly associated with vitamin D deficiency (either <50 nmol/L or <37 nmol/L) in men. Longer sleep (>8h) was associated with serum 25OHD <37 nmol/L (OR = 3.01, 95%CI 1.22–7.42) but not associated with serum 25OHD <50 nmol/L (Table 2).

In addition, having MetS in both vitamin D deficiency categories was associated with 2-fold significant adjusted risk of vitamin D deficiency (either <50 nmol/L or <37 nmol/L) in both women and men (women: OR = 2.04, 95%CI 1.31–3.17; OR = 2.15, 95%CI 1.11–4.17, respectively) (men: OR = 2.08, 95%CI 1.23–3.20; OR = 2.04, 95%CI 1.00–4.29, respectively) (Table 2).

As a significant interaction between sleep duration and MetS was detected in both women and men (p_interaction_ <0.001), these data were then stratified by short (≤6 h/day) or normal-long sleep duration (>6 h/day) (Table 3).

**Table 3:** Serum 25OHD <50 nmol/L or serum 25OHD <37 nmol/L risk stratified by duration of sleep (≤6h and >6h) in older Macanese (n = 204).

	%	Mean 25OHD (nmol/L)	25OHD <50 nmol/L		25OHD <37 nmol/L	
			Crude OR (95%CI)	Adjusted OR (95% CI)	Crude OR (95%CI)	Adjusted OR (95% CI)
**Women (n = 108)**						
Sleep ≤6 h (n = 35)						
MetS						
No (reference)	79	54 ± 17	1.0	1.0	1.0	1.0
Yes	21	42 ± 13*	3.34 (1.30–8.61)*	3.26 (1.10–9.64)*	5.36 (1.96–14.71)*	37.53 (7.06–199.61)*
Sleep >6 h (n = 73)						
MetS						
No (reference)	72	57 ± 19	1.0	1.0	1.0	1.0
Yes	28	53 ± 16	1.76 (1.09–2.84)*	1.41 (0.85–2.35)	0.83 (0.37–1.84)	0.64 (0.27–1.53)
**Men**^**b**^ **(n = 96)**						
Sleep ≤6 h (n = 30)						
MetS						
No (reference)	68	65 ± 22	1.0	1.0	1.0	1.0
Yes	32	64 ± 11	0.49 (0.22–1.12)	0.49 (0.20–1.21)	1.99 (0.60–6.57)	2.47 (0.69–8.80)
Sleep >6 h (n = 66)						
MetS						
No (reference)	84	63 ± 16	1.0	1.0	1.0	1.0
Yes	16	48 ± 14*	6.62 (3.49–12.56)*	5.22 (2.70–10.12)*	5.93 (2.63–13.35)*	4.86 (2.09–11.33)*

*p<0.05 for independent t-test and logistic regression compared to the reference group

^a^ Mutually adjusted for MetS, SES, PA and fish consumption

^b^ Mutually adjusted for MetS, SES, and PA; Data presented as mean+ SD where appropriate

On multivariable analysis, women with short sleep duration and having MetS had a significant 3-fold increased risk of vitamin D deficiency (serum 25OHD < 50nmol/L and <37 nmol/L) (OR = 3.26, 95%CI 1.10–9.64). The risk increased to 20-fold when severe vitamin D deficiency was investigated (OR = 37.53, 95%CI 7.06–199.61). It should be noted that only 35 women had short sleep duration, therefore, the small sample size led to the high OR and wide CI when we defined the cut-off for vitamin D deficiency as <37 nmol/L. In contrast, these associations were not found in those women with longer sleep. (Table 3).

The opposite effect was seen in men, where on multivariable analysis those with longer sleep and MetS had a 5-fold risk of vitamin D deficiency (serum 25OHD < 37nmol/L, OR = 4.86; 95%CI 2.09–11.33; serum 25OHD < 50 nmol/L, OR = 5.22; 95%CI 2.70–10.12) and a non-significant association between MetS and vitamin D deficiency for those with shorter sleep.

## Discussion

The current investigation of an older Macanese population found that both short and long sleep duration were associated with increased risk of vitamin D deficiency in women, but not in men.

Although there have been some clinical evidence linking sleep apnoea and vitamin D deficiency [38], few studies have investigated the association between sleep duration and circulating 25OHD levels or dietary vitamin D intake [53–66] (Table 4).

**Table 4:** Studies investigating the association between sleep duration and vitamin D (serum 25OHD levels or dietary intake).

Author, year	N	%F	Country/ Ethnicity & Study design	Sleep duration (mean ± SD)/ methods	25OHD (nmol/L) (mean ± SD or median [IQR])	Age (years) (mean / range)	Univariable association between sleep duration and 25OHD or dietary VD intake
Mason, 2016 [57]	218	100	USA; RCT	PSQI sub-score for sleep duration;	53 ± 15	60 ± 5	No difference in change in sleep duration between placebo and VD suppl. group;↔
Huang et al., 2013 [55]	28	64	USA; IN	4.5 ± 1.5; SR	46 ± 14	46 ± 11	↑ sleep duration after VD suppl. for 3 months;
Bertisch, et al., 2015 [53]	1721	55	USA; CS	6.5 ± 1.3; wrist actigraphy device;	63 ± 26	68 ± 9	↓ sleep duration in ↓ 25OHD<50nmol/L participants vs. 25OHD>75 nmol/L
Massa et al., 2015 [58]	2966	0	USA; CS	6.4 ± 1.2 h; wrist actigraphy device;	16% 25OHD<50;	>68	↓ sleep duration as ↓ 25OHD;
Piovezan, et al., 2017 [64]	657	56	Brazil; CS	46% had <6h sleep; polysomnographic;	60% 25OHD<75;	52 ± 9	↓ sleep duration ↓ 25OHD;
Darling et al., 2018 [61]	41	100	UK / CS	Sleep duration NA; Actigraphy & SR	South Asian 53 [51]; White Caucasian 83 [36];	39–75	No association between sleep duration and 25OHD; ↔
Beydoun, et al., 2014 [54]	2459	NA	USA; CS	37% had <6h sleep; SR	55 ± 3	20–85	↓ sleep duration ↓ 25OHD;
Kim, 2014 [56]	1614	54	Korea; CS	6.6 ± 1.6; 47% had <6h sleep; SR	49 ± 19	68 ± 5	↓ sleep duration ↓ 25OHD;
Song, 2016 [59]	2853	66	Korea; CS	7.1 ± 1.5 h/day; SR	42 [30–56]	72 ± 5	↓ sleep duration ↓ 25OHD in men
Darling et al., 2011 [60]	90	100	UK; CS	Sleep duration NA; PSQI	NA	NA	↓ sleep duration ↑ 25OHD;
Doo M., 2018 [62]	3757	56	Korea; CS	6.6 ± 0.05; SR	48 [range 10–134]	65–97	↓ sleep duration ↓ 25OHD;
Gong, et al., 2018 [65]	800	46	China, CS	9.17 ± 0.97; SR	56 ± 15	11 ± 2	↓ sleep duration ↓ 25OHD;
Grandner et al., [63]	4548	53	USA; CS	Very short (<5); Short (5–6); Normal (7–8); Long (≥9); SR	4.4 ± 5.0 mcg;	46 ± 17	↓ dietary VD intake in very short, short and long sleep duration compared to normal

Abbreviation: N, number of participants; F, female; 25OHD, 25-hydroxyvitamin D; SD, standard deviation; IQR, interquartile range; CS, cross-sectional; RCT, randomized control trial; IN, intervention; PSQI, Pittsburgh Sleep Quality Index; VD, vitamin D; suppl., supplementation; NA, not available; ↔, no association; ↑, increase; ↓, decrease; SR, self-report; Data presented as mean + SD where appropriate

Of the two studies [55, 57] that investigated vitamin D supplementation and change in sleep duration, one small study (n = 28) reported that chronic pain patients with serum 25OHD < 50nmol/L had a significantly shorter sleep duration than those with serum 25OHD between 50–75 nmol/L at baseline (3.73 hours vs. 5.33 hours); these chronic pain patients reported increased sleep duration after three months of vitamin D supplementation [55]. It should be noted, however, that our study had a higher average sleep duration than study by Huang *et al*., due to the difference in participant characteristics. The findings regarding the association between shorter sleep duration and increased risk of vitamin D deficiency were consistent. In contrast, the authors of the randomized control trail undertaken in overweight, vitamin D deficient postmenopausal women, did not observe improvements in sleep duration and sleep quality after 12 months of vitamin D supplementation [57].

Of the eleven observational studies investigating sleep duration, [53, 54, 56, 58–65], measured either by polysomnography [64], actigraphy [53, 58, 61] or as self-reported [54, 56, 59, 60, 62, 63], six large epidemiological cross-sectional studies reported a significant crude association between short sleep duration and decreased serum 25OHD levels [53, 54, 56, 58, 62, 64]. Three of these studies were from the USA and appeared to have slightly shorter average sleep duration and a higher mean 25OHD levels than our study [53, 54, 58]. Bertisch *et al*. reported a mean serum 25OHD level at 63 nmol/L and a mean sleep duration of 6.5 h/day [53]; Beydoun *et al*., reported a mean serum 25OHD level at 55 nmol/L and 37% had short sleep duration [54]; Massa et al., reported only 16% of the participants had vitamin D deficiency and the mean sleep duration was 6.4 h/day [58]. However, three Korean studies had similar sleep duration (mean sleep durations were 6.6, 7.1 and 6.6 h/day, respectively) and slightly lower mean 25OHD levels (all Korean studies reported mean serum 25OHD levels <50 nmol/L), although it should be noted that the population were older than in our study (all age >65 years) [56, 59, 62]. To our knowledge, study of the association between sleep duration and vitamin D status has been limited in Chinese population. A recently published paper from Ningbo, China, reported that vitamin D deficiency was associated with a two-fold likelihood of short sleep duration (< 9h) in school children [65]. In addition, a recent meta-analysis [66] reported that having low serum vitamin D levels was associated with increased risk of short sleep duration (pool estimate OR = 1.74; 95%CI 1.30–2.23). None of the included studies were in Chinese populations [66].

Of the three studies that investigated longer sleep duration [54, 56, 60], only one study (n = 93) reported an inverse correlation between sleep duration and serum 25OHD status (r = -0.0261) [60]. In addition, when investigating intake of dietary vitamin D, one large epidemiological US National Health and Nutritional Study reported significant lower dietary vitamin D intake mean associations between both long and short sleep [63].

Circadian entrainment research has indicated that the sleep-wake cycle is reset daily by retina-perceived light, controlled by a master clock in the suprachiasmatic nuclei of the hypothalamus that inhibits the release of melatonin [67]. One uncontrolled clinical trial reported that vitamin D supplementation in 1,500 patients with sleep disturbances resulted in normal sleep [68]. These authors thus linked sleep disturbances with vitamin D deficiency and suggested that 25OHD deficiency may be involved in circadian rhythm dysynchronization [68].

Metabolic syndrome has been reported to be associated with vitamin D status in a recent meta-analysis (Top vs. bottom tertile vitamin D levels, RR = 0.86, 95%CI 0.80–0.92; n = 6554) [69]. To our knowledge, this is the first time MetS has been associated with sleep duration (either short or long) and vitamin D deficiency. However, recently published data from an elderly Korean population showed a higher risk for obesity in those who were vitamin D deficient and had shorter sleep duration [62] (Table 4).

It is interesting that in our data, women with shorter sleep had a higher risk of vitamin D deficiency if MetS was present but men with MetS and longer sleep had a higher risk of vitamin D deficiency. We believe that this finding may be explained by the very high rate of vitamin D deficiency in women compared to men in this study population.

One explanation for the observations in the current study may be found in recent research that indicates the long duration of residence for the main vitamin D metabolite, 25(OH)D, in the circulation, is that it is taken up into muscle cells where it binds to vitamin D-binding protein (DBP) that is attached to cytoplasmic actin [70]. Thus, muscle acts as a storage center for 25(OH)D, where it is able to passage into and out of the muscle cells. There is evidence that this process is particularly useful for maintaining vitamin D status in the winter months when UV exposure is reduced. Furthermore, it has been proposed that this mechanism can be negatively affected when muscle function is compromised by inactivity or malnutrition. Sleep duration is well known to impact muscle function [71–73]. Taken together, it is likely that sleep requirements for adequate muscle function also affect the storage of vitamin D and if, over long periods of time, a person has disturbed sleep, a risk of vitamin D deficiency develops. Of note, the significant differences we observed here in the 25(OH)D mean values suggest physiological changes in the mechanisms controlling blood 25(OH)D levels, rather than indications of relative sufficiency or deficiency of functional vitamin D. Indeed, one study in rats has shown that the relative levels of muscle-active hormones is related to sleep [74]. Thus, a future follow-up study to further enhance the understanding of the mechanism behind our current findings would be to measure various blood hormone levels in participants with sleep deprivation and determine any correlations in these endocrine changes to changes in muscle handling of 25(OH)D.

The limitations of the current study include the cross-sectional study design and small sample size (especially when stratified). As with all cross-sectional studies, it is impossible to disentangle causality. Central obesity and fasting blood glucose were not able to be included in our definition of metabolic syndrome, as these measurements were not taken at the study visits. To address this, a BMI cut off at 24 kg/m2 was applied in the current study. Several studies from China have suggested the optimal cut-off points to predict the presence of metabolic syndrome in adults were BMI between 23–24 kg/m^2^ [75–78]. In addition, information on other sleep-related disturbances or disorders, including sleep apnoea, were not collected in our study. Several studies have reported that sleep apnoea was associated with lower serum 25OHD levels [79–87]. Furthermore, it remains possible that the associations between sleep and vitamin D status may be due to associations with poor health [88], including unmeasured confounding variables such as depression [89] and musculoskeletal pain [39] or inflammation [90]; these unmeasured confounding variables may differ by sex especially in Chinese populations. The strengths of this investigation are the representativeness of the population and that many risk factors for CVD were individually measured, enabling us to calculate MetS.

## Conclusion

Both short and long sleep duration were associated with vitamin D deficiency in the older Macao women. Notably, women with shorter sleep and MetS had a very high risk of vitamin D deficiency. While the underlying unmeasured factors determining sleep duration may also contribute to MetS risk and vitamin D levels, further studies especially stratified by sex in Asian populations may provide insight into the causality of these associations.

## Supporting information

S1 Data (XLSX)
